# Supplementary material for: Climate Action at Public Health Schools in the European Region
Source: Int J Environ Res Public Health. 2021 Feb 5;18(4):1518. doi: 10.3390/ijerph18041518 (PMC7914969; doi:10.3390/ijerph18041518)
Supplement: Supplementary file 1 [file ijerph-18-01518-s001.pdf]

International Journal of
Environmental Research and Public Health

MDPI

Article

Climate Action at Public Health Schools in the European Region

Rana Orhan [1,2,3*]

Supplementary Material S1: Survey Questions

Climate action by public health schools in the European region

Note: The participant information page and the consent form have not been included in the supplementary material.

Start of Block: Participant details

Q12 Name
Q13 Title
Q14 Email address
Q15 Name of your school

End of Block: Participant details

Start of Block: Climate action

This part of the questionnaire focuses on climate action taken by the public health school.

Vision and strategy

1.1 Is climate action included in the school's vision?

- Yes (1)
- Yes, but only implicitly via the university's vision (2)
- No, but there are concrete plans to do so (3)
- No (4)

Skip To: 1.3 If 1.1 = No

Skip To: 1.3 If 1.1 = No, but there are concrete plans to do so

1.2 Which statement is best applicable to the school's vision?

- The vision on climate action is implicitly applied within the school (e.g. via the university's vision). (1)
- The school has an explicit vision on climate education, which is being applied within the majority of the programme. (2)
- The vision on climate education is visible in the profiling of the school or the educational programme. The vision is being kept updated. (3)
- The school actively collaborates with the professional field and centres of expertise in developing their vision on climate action and its periodic updating. (4)
- The school is a recognised pioneer in translating the concept of climate action to the educational domain. (5)
- Other (please specify) (6) ____

1.3 Is climate action included in the school's strategy?

- Yes (1)
- Yes, but only implicitly via the university's strategic plans (2)
- No, but there are concrete plans to do so (3)
- No (4)

Skip To: 1.5 If 1.3 = No

Skip To: 1.5 If 1.3 = No, but there are concrete plans to do so

1.4 Which statement is best applicable to the school's strategy?

- The strategy on climate action is implicitly applied within the school (e.g. via the university's strategic plans). (1)
- Different actors within the school formulate a strategy and objectives for their own activities. (2)
- The school has an explicit view on climate action translated to concrete objectives within several policy areas. (3)
- The school periodically evaluates and reflects the realisation of the concrete objectives. The evaluation takes place in coherence with the vision on climate action. (4)
- The school actively collaborates with the professional field and centres of expertise in developing its strategies on climate action and its periodic updating. (5)
- The school is a recognised pioneer in translating the concept of climate action to several policy areas. (6)
- Other (please specify) (7) ____

1.5 If you are willing to share your strategy, please email Rana Orhan at ranaorhan@gmail.com. If available online, please provide the URL: ____

Personnel and resources

1.6 Which statement is best applicable to the school's personnel?

- A few employees have knowledge in the field of climate action in the educational domain. The school provides the opportunities to execute individual educational initiatives. (1)
- Developing expertise on climate action is part of the personnel's development plan. (2)
- Expertise in the field of climate action is broadly present in the school. This expertise is being kept updated systematically. (3)
- Knowledge and expertise at knowledge institutions and the professional field are part of the education programme. Expertise is being developed in collaboration with relevant partners. (4)
- Employees of the school and the linked institutions are leading in climate action in the educational domain. (5)

- Other (please specify) (6) ____
- None of these (7)

1.7 Which statement is best applicable to the school's networks?

- Individual employees have contact with the professional field and/or centres of expertise in the field of climate action. (1)
- The school has contact with companies and centres of expertise in the field of climate action. (2)
- The school develops its network on the basis of the desired exchange of knowledge and expertise about climate action and the training thereof; it is regularly checked on this. (3)
- The school provides education and executes research in the field of climate action, together with the professional field and centres of expertise. The content of education and the research agenda are being developed and evaluated together. (4)
- The network of the school in the field of climate action consists of a broad spectrum of societal actors. The school plays a recognised leading role in its environment. (5)
- Other (please specify) (6) ____
- None of these (7)

1.8 Which statement is best applicable to the school's culture?

- The program takes measures on a number of points to achieve common values for climate action. (1)
- The school has a coherent policy aimed at developing common values around climate action and the appropriate behaviour of students and teachers. (2)
- The school is recognised by a culture in which values linked to climate action are central. This culture is actively being maintained. (3)
- The school develops common values for climate action with its partners and appropriate behaviour of students and graduates. (4)
- The program fulfils a recognised societal role and is an inspiring example for others. (5)
- Other (please specify) (6) ____
- None of these (7)

1.9 Which statement is best applicable to the school's physical environment?

- On a number of points, the school takes measures with regard to its physical environment. (1)
- There is a concrete policy aimed at reducing the ecological footprint of the school and at contributing to environmental restoration. (2)
- The school works systematically to reduce its ecological footprint and makes a concrete contribution to restoring the living environment. (3)

- Together with its partners, the school takes measures aimed at reducing its ecological footprint and its partners and at contributing to environmental restoration. (4)
- The school has innovative solutions for reducing the ecological footprint and for contributing to environmental restoration and is an example for others. (5)
- Other (please specify) (6) ____
- None of these (7)

1.10 Which measures does the school take to reduce its ecological footprint?

- o A net zero carbon building (1)
- o Fossil fuel divestment or a fossil fuel free school (2)
- o Green travel policy (3)
- o A single-use plastic free building (4)
- o Avoiding waste (5)
- o Improving waste management (6)
- o Increase re-use, repair or recycling (7)
- o Using waste from some production processes as a resource in others (8)
- o Increase biodiverse green space (9)
- o Promoting efficient & economical water supply & use (10)
- o Raising awareness among students (not by formal education) (11)
- o Raising awareness among personnel (12)
- o Providing students with the opportunity to be involved in sustainability (13)
- o Providing personnel with the opportunity to be involved in sustainability (14)
- o Other (please specify) (15) ____
- o ⊗None of these (16)

1.11 Is your school accepting donations and grants from fossil fuel companies or other environmentally destructive companies?

- Yes (1)
- No (2)
- I do not know (3)

1.12 Which statement is best applicable to the school's communication?

- Communication about climate action is incidental and involves separate activities. (1)
- Internal and external communication run parallel to the activities carried out on climate action. (2)
- The school has and implements an explicit communication policy on climate action. (3)
- Communication about climate action takes place in coordination with partners and are carried out jointly. (4)
- The school is the communication platform for various parties in the field of climate action in the educational domain. (5)

- Other (please specify) (6) ____
- None of these (7)

Education and research

1.13 Which statement is best applicable to the school's education?

- The perspective of climate change in research and practical assignments depends on the individual teacher or student. (1)
- Each student carries out a practical assignment at least once from the perspective of climate change. (2)
- When carrying out assignments in the professional field, students use the perspective of climate change. (3)
- Together with the professional field and centres of expertise, the school implements projects aimed at innovations in climate action in professional practice. (4)
- The school plays a leading role in multidisciplinary innovation projects in the field, together with societal organisations, educational institutions and centres of expertise. (5)
- Other (please specify) (6) ____
- None of these (7)

1.14 Which statement is best applicable to the school's research?

- The presence of climate change aspects in research projects depends on the individual teacher or student. (1)
- The school ensures that every student uses an integral perspective on climate change in at least one study. (2)
- Teachers and students have an integrated perspective on climate change in answering their research question. (3)
- The school is involved in research programmes with other centres of expertise and research institutions aimed at innovations in climate action. (4)
- The school plays a leading role in multidisciplinary research programmes together with societal organisations, educational institutions and centres of expertise. (5)
- Other (please specify) (6) ____
- None of these (7)

Results

1.15 Which statement is best applicable to the school?

- Research and/or graduation projects occasionally lead to innovative solutions for issues associated with climate change. (1)
- The school stimulates innovative research and/or graduation projects to come up with solutions for issues associated with climate change. (2)

- Research and education are systematically aimed at developing innovations for issues associated with climate change in professional practice and research. (3)
- In research and education, the school collaborates with external partners to develop innovations in the knowledge domain and professional practice. (4)
- The innovative solutions that have been realised in research and education of the school, together with the partners, are known in the wider environment. (5)
- Other (please specify) (6) ____
- None of these (7)

End of Block: Climate action

Start of Block: Climate education

This part of the questionnaire is from the Global Consortium on Climate & Health Education (GCCHE).

The GCCHE aims to develop a global community among health professions schools interested in establishing or expanding climate-health education. The GCCHE is being led by the Columbia University Mailman School of Public Health, home to the US' first academic Climate and Health Program, with financial support from the Rockefeller Foundation[1]. In order to establish a baseline for charting future progress, the GCCHE conducted this part of the survey on the current state of climate-health curricula internationally among Consortium members (in the period 2017-2018).

2.1 Does your school offer climate-health education?

- Yes (1)
- No (2)

Skip To: 2.17 If 2.1 = No

2.2 What climate-health education does your school offer? (Please select all that apply)

- o Climate-health session as part of non-required course (1)
- o Climate-health session as part of required core course (2)
- o Climate-health standalone elective course (3)
- o Climate-health standalone required course (4)
- o Climate-health masters or certificate program (5)
- o Climate-health doctoral program (6)
- o Climate-health post-doctoral positions (7)

Display This Question:

If 2.2 = Climate-health standalone elective course

Or 2.2 = Climate-health standalone required course

2.3 If your school offers a standalone course on climate-health, what is its title, and how many credits are received upon completion?

---

[1] During the time of submission, this is not the case anymore.

- Title: (1) ____
- Credits: (2) ____

Display This Question:

If 2.2 = Climate-health standalone elective course

Or 2.2 = Climate-health standalone required course

2.4 If your school offers a standalone course on climate-health, what teaching methods are used? (Please select all that apply)

- Labs (1)
- Lectures (2)
- In-class exercises (3)
- Online tutorials or MOOCs (Massive Open Online Courses) (4)
- Internships outside the classroom (5)

Display This Question:

If 2.2 = Climate-health masters or certificate program

2.5 If your school offers a masters or certificate program in climate-health, what is its title, and how many credits are needed for its completion?

- Title: (1) ____
- Credits: (2) ____

Display This Question:

If 2.2 = Climate-health masters or certificate program

2.6 If your school offers a masters or certificate program in climate-health, what teaching methods are used? (Please select all that apply)

- Labs (1)
- Lectures (2)
- In-class exercises (3)
- Online tutorials or MOOCs (Massive Open Online Courses) (4)
- Internships outside the classroom (5)

2.7 What are the main goals of your overall climate-health curriculum?

2.8 How long have the climate-health education teachings been in place?

2.9 Has your school received evaluations from students on their experience of and/or satisfaction with the climate-health teachings?

- Yes (1)
- No (2)

Display This Question:

If 2.9 = Yes

2.10 If you are willing to share the results of the evaluations, please email Rana Orhan at ranaorhan@gmail.com.

2.11 Does your school assess students' climate-health knowledge?

- Yes (1)

- No (2)

Display This Question:
If 2.11 = Yes

2.12 If "Yes", how is climate-health knowledge assessed? (Please select all that apply)
- o Quizzes (1)
- o Exams (2)
- o Papers (3)
- o Capstone (4)
- o Thesis (5)
- o Dissertation (6)

2.13 If available, please provide the URL to your climate-health curriculum website: ________________________________

2.14 Does your school offer Planetary Health modules, courses, or programs?
- Yes (1)
- No (2)

2.15 Are your Planetary Health and climate-health modules or programs linked or integrated?
- Yes (1)
- No (2)

2.16 Does your school currently have any partnerships on climate change and human health? (Please select all that apply)
- o Yes, with another academic institution on training (1)
- o Yes, with another academic institution on research (2)
- o Yes, with a non-academic institution (business, government, NGO, etc.) (3)
- o Yes, with a funder (4)
- o ⊗No (5)

2.17 Are any climate-health offerings under discussion to add? (Please select all that apply)
- o Session as part of non-required course (1)
- o Session as part of required core course (2)
- o Climate-health standalone elective course (3)
- o Climate-health standalone required course (4)
- o Climate-health masters or certificate program (5)
- o Climate-health doctoral degrees (6)
- o Climate-health post-doctoral positions (7)
- o ⊗Nothing being considered (8)

2.18 Have you received a positive response to adding climate-health curriculum? (Please select all that apply)

- o Yes, from students (1)
- o Yes, from faculty (2)
- o Yes, from administration (3)
- o No, have not received a positive response (4)
- o Other (please specify) (5) ____

2.19 Have you encountered any challenges in trying to institute climate-health curriculum? (Please select all that apply)

- o Yes, lack of interest or demand from students (1)
- o Yes, administration or other skepticism about climate-health science (2)
- o Yes, lack of funding/time to support its development (3)
- o Yes, lack of available staff time to work on its development (4)
- o Yes, no available space in the core curriculum (5)
- o Yes, lack of teaching materials and staff expertise (6)
- o Yes, competing institutional priorities/politics (7)
- o No challenges (8)
- o Other (please specify) (9) ____

2.20 What have you found helpful in instituting or developing climate-health curriculum? (Please select all that apply)

- o Interest from students (1)
- o Interest from faculty (2)
- o Interest from administration (3)
- o Support from Board members (4)
- o Support from donor (5)
- o Other (please specify) (6)

End of Block: Climate education

Start of Block: Last part

3.1 Please feel free to share any comments here, or send them directly to the contact persons as mentioned on the final page.

____

____

____

End of Block: Last part
